# Inflammation and Cell Death of the Innate and Adaptive Immune System during Sepsis

**DOI:** 10.3390/biom11071011

**Published:** 2021-07-10

**Authors:** Christina Nedeva

**Affiliations:** Department of Biochemistry and Genetics, La Trobe Institute for Molecular Science, La Trobe University, Bundoora, VIC 3086, Australia; C.Nedev@latrobe.edu.au

**Keywords:** inflammation, infection, sepsis, immune activation, cell death

## Abstract

Sepsis is a life-threatening medical condition that occurs when the host has an uncontrolled or abnormal immune response to overwhelming infection. It is now widely accepted that sepsis occurs in two concurrent phases, which consist of an initial immune activation phase followed by a chronic immunosuppressive phase, leading to immune cell death. Depending on the severity of the disease and the pathogen involved, the hosts immune system may not fully recover, leading to ongoing complications proceeding the initial infection. As such, sepsis remains one of the leading causes of morbidity and mortality world-wide, with treatment options limited to general treatment in intensive care units (ICU). Lack of specific treatments available for sepsis is mostly due to our limited knowledge of the immuno-physiology associated with the disease. This review will provide a comprehensive overview of the mechanisms and cell types involved in eliciting infection-induced immune activation from both the innate and adaptive immune system during sepsis. In addition, the mechanisms leading to immune cell death following hyperactivation of immune cells will be explored. The evaluation and better understanding of the cellular and systemic responses leading to disease onset could eventuate into the development of much needed therapies to combat this unrelenting disease.

## 1. Introduction

Presently, sepsis kills eleven million people each year whilst disproportionally affecting infants, the elderly, pregnant women, and people from low-income countries [1]. Recent studies have shown that one in every five deaths world-wide are attributed to sepsis, double the number of deaths accredited to this disease compared to estimates from previous years [2]. Despite developments in our understanding of the pathogenesis of sepsis, it continues to be a world-wide health challenge. This is mainly due to the associated organ dysfunction, protracted inflammation, immune suppression and predisposition to secondary infection, which can all lead to premature death [3].

In general, sepsis is considered to be a biphasic disease, however, both phases can occur synchronously [4]. The initial hyperinflammatory phase, also known as the “cytokine storm”, is described by the overwhelming release of inflammatory molecules by the innate immune system, potentially leading to the destruction of tissue [5]. Shortly after this spike in inflammation, the immune system dampens, leading to a hypo-inflammatory state. Here, the immune system exhibits exhaustion and death of cells from both lymphoid and myeloid lineage, leaving patients immunocompromised [6]. The mass depletion of immune cells leaves patients vulnerable to secondary infection, typically caused by nosocomial opportunistic pathogens such as *Acinetobacter baumannii* (22.2% of cases) *Pseudomonas aeruginosa* (10.3% of cases), *Candida albicans* (candidiasis; 8.5% of cases) and viruses (>1% of cases) [7,8,9,10,11,12]. Secondary infections are typically acquired 48 h proceeding the initial infection, suggesting that immune paralysis peaks during this time, however this varies between cases and is dependent on many factors, i.e., patient comorbidities [13]. Studies that have specifically investigated the incidence and impact of secondary infection on clinical outcome have shown that opportunistic fungi and bacteria significantly increase in the later phase (>15 days) of sepsis when compared to the early phase (<6 days) [14]. Additionally, other studies have revealed that sepsis patients who died 3 days or more after ICU admission had acquired secondary infections [15]. Moreover, depending on the severity of the infection causing sepsis, the immune system may never fully recover, leaving patients burdened with prolonged immune disfunctions [16]. As this dysfunction extends to both the cells of the innate and adaptive immune system, it is critical to understand the molecules and mediators that act to disrupt normal cellular responses as well as causing their demise.

Multiple novel therapies to combat sepsis have been developed, however, deaths due to sepsis continue to increase. Hence, antibiotic therapy, resuscitative strategies, blood glucose-level management and ventilator use continue to be the only validated actions against this disease [17,18]. The lack of concrete therapies against sepsis exposes gaps in our knowledge. These limitations are evidenced by the multiple drug trials that have failed in the past, and in some instances increased mortality, largely due to efforts to target inflammation [19]. It is now known that a surge in inflammatory molecules signaling the critical “kick-start” to the immune system is necessary to fight invading pathogens, especially during culturable sepsis [4,20]. Therefore, targeting inflammation could be detrimental rather than beneficial. Additionally, it is well documented by numerous studies that the principal driver of sepsis is the hosts response to infection, rather than the invading organisms themselves [21]. Therefore, understanding the role immune cells play, and how immune cell death occurs during sepsis is essential for the future development of therapeutic tools.

## 2. Immune Activation and Sepsis

During the early stages of severe infection, necrotic tissue and microbes release destructive substances into the system, which consist of damage-associated molecular patterns (DAMPs) and pathogen-associated molecular patterns (PAMPs), respectively. These harmful pyrogens cause the rapid activation of a series of membrane receptors known as pattern recognition receptors (PRRs), which include toll-like receptors (TLRs), expressed by cells of the innate immune system [22]. Shortly after the detection of an infectious agent, first line defenders including macrophages, dendritic cells and neutrophils expressing these receptors become highly proliferative in an attempt to clear the overwhelming infection as quickly as possible [23]. Following these events, the adaptive immune system takes effect leading to the activation of T helper and cytotoxic T cells via T cell receptor (TCR) activation. Subsequent differentiation and proliferation of these cells leads to a highly specific adaptive immune response [24].

### 2.1. Acute Innate Immune Response

The innate immune response is triggered promptly upon the invasion of a foreign body or antigen. In the case of a gram-negative bacterial infection, one PRR that is activated is TLR4 by lipopolysaccharide (LPS) molecules present on the surface of the bacteria [23]. Following activation, TLR4 forms a complex with CD14 and MD2, triggering a cascade of intracellular signaling events leading to the production of transcription factors, mainly NF-κB, Ap-1 and IRF3 [25,26]. Specific cells equipped with pathogen detection components including endothelial cells, dendritic cells, natural killer cells monocytes in the blood and macrophages in the tissues, go on to produce and release an abundance of inflammatory mediators once activated. Critical inflammatory factors include IL-1β, IL-2, IL-6, TNF-α and chemokines such as prostaglandins, histamine, and IL-8. These molecules target vascular endothelial cells, causing the release of nitric oxide (NO) into the system, in turn, increasing vascular permeability [27]. In parallel, circulating neutrophils expressing functional receptors such as CXCR1 and CXCR2 receive signals from activated antigen presenting cells (APCs) at the site of infection, alerting them of the foreign body [28]. At this point, circulating neutrophils mobilise and adhere to the epithelial membrane via L-selectin and integrins in the high affinity state [29]. Here, neutrophils start to infiltrate the leaky vessels at the site of infection via the process of extravasation. When in the tissue, neutrophils execute their effector functions, which involves degranulation, phagocytosis of the pathogen and NET formation [30]. Other invading APCs also partake in phagocytosis and presentation of foreign peptides upon MHC class II molecules to facilitate clearance [31]. At the local site the coagulation cascade ensues with the upregulation of clotting factors promoting platelet aggregation. These processes further amplify the inflammatory response until the infection is resolved [32].

In the case of viral infections, including respiratory infections such as influenza, coronavirus, respiratory syncytial virus (RSV) and rhino viruses, innate responses differ to that of bacterial infections. Viral PAMPs activate PRRs such as TLR7 which sense single stranded RNA (ssRNA) [33]. Following PRR activation, downstream signaling events induce transcription of NF-κB and IRF, upregulating proinflammatory and anti-viral responses. The interferon (IFN) system is key in supporting antiviral immunity as it affects virus replication and downstream antiviral immunity [34]. These responses are mediated by Type I (IFNα/β) and Type III (IFNλ) IFNs, whereas Type II INFs (IFNγ) promotes immune cell responses. Immune cells such as alveolar macrophages (most abundant pulmonary leukocyte) and dendritic cell subsets are some of the first cells to respond to signal released by virally infected cells. Neutrophils (being the most abundant polymorphonuclear cell population observed un the bronchioalveolar lavage) similarity assist in pathogen eradication by phagocytosis and NET formation [35].

During sepsis, the local response becomes systemic leading to widespread infection, and therefore inflammation, throughout the body [36]. This widespread vasodilation leads to hypotension and tissue oxygen deprivation and the associated disseminated intravascular coagulation (DIC) causes coagulopathy. Specifically, during viral sepsis, altered endothelial cell (EC) physiology and the dysfunction of the lung tissue barrier, leads to endothelial activated cytokine storm [37]. Here, IFN-α produced by ECs serves to enhance immune responses by activating dendritic cells, monocytes, and NK cells. These cells themselves exacerbate immune responses by releasing consequential cytokines into the system, such as IFN-γ [38]. Additionally, sepsis-related lung injury appears to be caused by the activation of endothelial TLR-4. Activation of EC TLR-4 leads to the recruitment of damaging neutrophils to the lungs [39]. Hyperactivated neutrophils produce an abundance of reactive oxygen species (ROS), which have deleterious effects on the surrounding tissues, and this causes changes to cellular metabolism. In addition, the overwhelming cytokine response includes the excessive release of IL-1β by tissue resident macrophages. Dysregulated IL-1β release acts to “hyper-activate” macrophages in an autocrine way, leading to a further exacerbated inflammatory response. This process known as macrophage activation syndrome (MAS) has been shown to occur in a small percentage of severe sepsis cases, however, our understanding of the frequency and occurrence of MAS during sepsis remains unclear [40]. The effect of these collective changes, among others, increases the severity of sepsis, which can lead to multiple organ failure and mortality [41,42].

### 2.2. Acute Adaptive Immune Response

While the first line defenders are combating infection, T and B cells of the adaptive immune system have time to fine tune antigen-specific responses. The activation of CD4^+^ T cells leads to the polarisation of these cells into specific T helper (Th) subsets including Th1, Th2 and Th17 [43]. Th1 cells are critical for the expansion of memory T cells via IL-2 secretion and for initiating CD8^+^ T cell activation. Also, Th1 primarily produce proinflammatory cytokine IFN-γ to further promote phagocytosis and eradication of microbes [44]. Th2 release IL-4 and IL-5, which act to induce class switching of B lymphocytes, and IL-10 to resolute inflammation [24]. The balance of crosstalk between Th1 and Th2 is important for clearing infection. However, when this balance is disrupted, such is the case with sepsis, this can lead to secondary infections, autoimmune diseases, and viral reactivation [45]. Under normal conditions, both Th1 and Th2 lymphocytes naturally produce IL-3, which stimulates the production granulocytes, dendritic cells and macrophages. Recently, IL-3 has received attention as a potential therapeutic target due to its myelopoietic functions during server infection [46]. Effector Th17 cells specifically act in response to bacteria and extracellular fungal pathogens, and in doing so produce cytokines such as TNF-α, IL-17 and IL-22 [47].

Furthermore, CD8^+^ T cells aid in clearing infection and are responsible for the generation of memory CD8^+^ T cells in response to infection. The cytotoxic functions of CD8^+^ T cells are elicited when CD8^+^ cells bind cognate antigen in the presence of cytokines and co-stimulatory molecules. This event leads to rapid proliferation and expansion, granting them effector functions including the release of TNF-α and IFN-γ, and cytotoxic functions [48,49].

Regulatory T cells (T_regs_), CD4^+^ and CD25^+^, which represent less than 10% of the total CD4^+^ T cell population in the lymph nodes and circulation, have a critical role in immune cell modulation in both steady-state and disease settings. T_regs_ respond to infection by suppressing excessive immune responses elicited by other cells of the adaptive immune system, in-turn, dampening inflammation. They also function to sustain self-tolerance through the secretion of TGF-β and IL-10, as well as targeting DC antigen presentation [50].

The unconventional subset of T cells, known as γδ T cells, make up approximately 0.5–5% of the proportion of cells in the circulation compared to other populations of T cells. They possess a distinctive TCR on their surface composed of γ and β chains as opposed to the classic α and β chains. They work to maintain immune homeostasis in the lung and intestinal epithelium, where they protect against pneumonia and gut infections, respectively. They mediate these protective functions against invading pathogens via the release of IL-17 and IFN-γ [51].

B cells are at the center of the adaptive immune response where they mediate the production of antigen-specific antibodies against specific pathogens. They have additionally been shown to enhance innate responses during sepsis through IFN receptor stimulation [52]. However, similarly to T cells, B cells also become dysregulated and exhausted as infection spreads to become systemic. In sepsis, these processes become severely disrupted, and therefore, cells of the adaptive immune system are unable to mount the appropriate defense response against the infection [50].

## 3. Immune Suppression and Cell Death during Sepsis

Under normal circumstances, following the resolution of an infection, the patient’s body will return to homeostasis. This process can take effect days after pathogen exposure and involves dampening of the cytokine cascade [5]. Leukocytes produce cytokines such as IL-10 which suppress proinflammatory cytokines IFN-γ and IL-6. This also stimulates the production of soluble IL-1 and TNF-α receptor antagonists, which act to neutralise cognate receptors. Intrinsically, antigen debris, damaged cellular structures and perturbed proteins are targeted for lysosomal degradation [53]. These cumulative events act to reduce cellular activation and inflammation.

Proceeding the acute inflammatory response caused by severe infection where patients survive but the immune system fails to appropriately resolve itself, systemic immune cells undergo a variety of phenotypic changes. The production, effector cell function and survival of cells of the innate and adaptive immune system are directly affected, leading to ubiquitous immune suppression. Consequently, the persistence and recurrence of secondary infections transpires, which causes an increase in chronic mortality rates amongst sepsis patients [54,55].

### 3.1. Chronic Innate Immune Response

In the later stages of sepsis, the once overactivated immune system progresses into an anti-inflammatory state. Here, innate and adaptive immune cells are severely dysregulated, leading to tissue damage and cell death [6].

**Figure 1:**
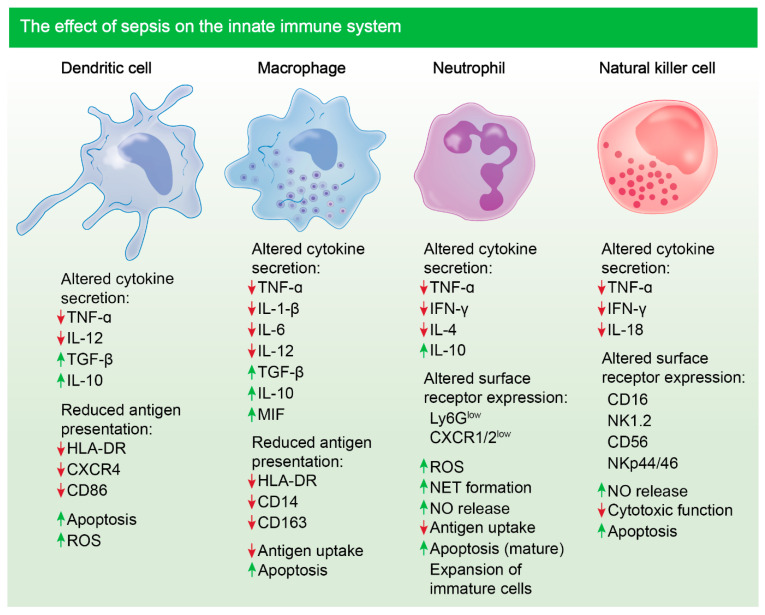
Sepsis-induced immune dysregulation and suppression of innate immune cells. Altered cytokine secretion and reduced antigen presentation is notable in sentinel dendritic and macrophage cells following sepsis in patients. Cytokines such as TNF-α are reduced and cytokines such as IL-10 are upregulated [73,74,75,76,77]. Altered cytokine secretion and surface receptor expression is notable in neutrophils and NK cells post-sepsis [78,79,80]. Marked apoptosis of most innate immune cells is evident [81]. Mature neutrophils undergo apoptosis during sepsis, however expansion of immature, damaging neutrophils occurs [7].

Neutrophils, which are the most prevalent leukocyte released into the circulation from the bone marrow, are essential for pathogen eradication [56]. They can act as APCs to synchronise the innate and adaptive immune response during infection [57]. Under steady-state conditions, neutrophils are short-lived with a turnover rate of 6–8 h in the circulation [58]. However, during sepsis, mature neutrophils become apoptotic, leading to an expansion of immature neutrophils owing to a delayed apoptotic response (Figure 1) [7]. The persistence of dysregulated immature neutrophils leads to extensive tissue damage as they have increased adhesive properties, allowing them to readily invade tissue and execute damaging functions such as oxidative burst [59]. Studies have shown that during sepsis, surviving impaired neutrophils persist due to the downregulation of pro-apoptotic proteins, such as Bim and caspases, with concurred upregulation of pro-survival proteins such as BCL-xL [7,60]. Similar studies in mice have also revealed that death of mature neutrophils, during sepsis, is mediated by triggering receptor expressed in myeloid like-4 (Treml4), leading to the prevalence of dysfunctional neutrophils and reduced survival of mice following secondary infection [7]. These findings highlight the importance of neutrophils during sepsis and how their dysregulation negatively impacts survival outcome.

Notably, monocyte and macrophage populations undergo various changes during sepsis including endotoxin tolerance. Under pathological conditions, monocytes have a decreased capacity to release inflammatory mediators, such as TNF, IL-1, IL-6 and IL-12, in response to infection, which is consistent with this phenomenon (Figure 1) [61]. On the contrary, their ability to release inhibitory cytokines such as IL-10 is unimpaired and, in some cases, enhanced. Studies have shown that blockade of IL-10 can alleviate immune suppression and increase survival of mice with sepsis [62]. Additionally, reduced expression of human leukocyte antigen, HLA-DR, is associated with poorer outcome following sepsis, as antigen presentation is compromised. The expression level of HLA-DR also correlates with the responsiveness of monocytes following expression and can be used as a surrogate marker [63].

One of the more potent APCs that have an important role in pathogen recognition and linking the innate and adaptive immune systems during infection are DCs. Notably, reduction in DC numbers, following sepsis, is associated with a poorer survival outcome in the clinic (Figure 1) [64]. In mouse models of sepsis, it has been shown that DCs are markedly reduced in mesenteric lymph nodes and the spleen following cecal ligation puncture [65]. The functionality of DCs is also impaired following severe infection, with a reduction in HLA-DR expression and an increase in IL-10 release [66]. These cells essentially become tolerogenic, whilst further dampening inflammation, respectively. Additionally, compromised DCs lack the ability to induce allogeneic activation of T cells, hence, effector T cell response is lost, leading to added immune suppression [67]. Studies have shown that blocking sepsis-induced apoptosis of DCs enhances survival, suggesting that preventing DC death could be a putative treatment avenue for sepsis [68].

During microbial sepsis, the primary producers of IFN-γ are NK cells. When stimulated by bacterial antigens, IL-12 and IL-18, produced by monocytes, cause NK cells to release IFN-γ, which largely activates macrophages [69]. However, a direct link between the release of NK cell, released IFN-γ and macrophage activation is yet to be established in the context of sepsis, although, it has been shown that NK cell numbers do markedly reduce during sepsis and those cells remaining have impaired cytokine response to endotoxin. Most importantly, the increase in apoptosis of NK cells during sepsis leads to a decrease in cell number for weeks post-initial septic insult [70,71]. Cytotoxic functions of NK cells are also compromised, contributing to immune suppression and increased patient risk to secondary infection during sepsis (Figure 1) [72].

### 3.2. Chronic Adaptive Immune Response

**Figure 2:**
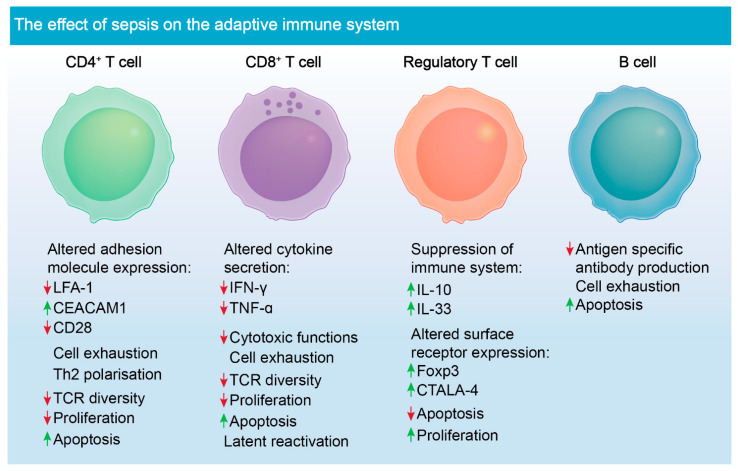
Sepsis-induced immune dysregulation and suppression of adaptive immune cells. Altered adhesion molecule expression is noted in CD4^+^ T cells following sepsis [95,96]. CD4^+^ cells lack appropriate functions post-sepsis due to down-regulation of TCRs. They also undergo marked apoptosis, as do CD8^+^ T cells. CD8^+^ T cells have altered cytokine secretion and decreased cytotoxic functions post-sepsis [6,97]. T_regs_ are the only known lymphocyte to proliferate during sepsis and exacerbate immune suppression during disease pathogenesis [98,99]. The role of B cells is not well defined in the context of sepsis; however, antigen-specific antibody production is compromised, and the cells become increasingly apoptotic during sepsis [50].

Lymphocytes, such as T cells, are a key population of cells critical for mounting successful adaptive immune response in all aspects of infection. CD4^+^ T cells specifically have crucial roles in modulating effective immune responses through the release of cytokines as well as eliciting cellular communication (Figure 2) [82]. These cell types rapidly expand and divide into several subsets such as Th1, Th2 and Th17, when exposed to peptide antigens. During sepsis, CD4^+^ T cells undergo the greatest volume of apoptosis, and this event strongly correlates with patient survival [3,83]. Studies have shown that attrition of CD4^+^ and CD8^+^ T cells causes marked lymphopaenia, causing aberrations in clonal expansion and an increased chance of viral reactivation in affected patients (Figure 2) [84]. Given the extensive cell death leading to the depletion of these T cells, immunostimulatory molecules such as IL-7, produced by stromal cells, have been investigated. This cytokine was shown not only to prevent cell death, but also leads to diminished immunosuppression caused by uptake of apoptotic cells by phagocytic cells [85]. Additionally, CD4^+^ and CD8^+^ have reduced TCR diversity post-sepsis episode, which further increases the risk of developing secondary infection [86].

Treg cells naturally have suppressive functions to help maintain tolerance to self-antigens and to prevent autoimmune disease [87]. These suppressive functions, however, become dysregulated during sepsis, which in turn, is detrimental to the activation and proliferation of effector T cells during and post-infection [88]. Release of inhibitory cytokines by dysregulated T_regs_ also leads to the inhibition of monocytes and neutrophils, having a negative impact on pathogen clearance (Figure 2) [89]. T_regs_ are less susceptible to apoptosis during sepsis, unlike other T cell populations, and more of them are circulating during disease pathogenesis [90]. However, depletion of T_regs_ using monoclonal antibody did not improve survival in mouse models of sepsis [91]. This finding suggests that their role in immune suppression during sepsis may be redundant and a consequence of other immunological deficits.

Under homeostatic conditions, B cells differentiate into plasma cells or memory B cells [92]. They facilitate humoral responses, and they participate in the activation of T cells. B cells main function, antibody generation and antigen presentation to T cells is, however, severely compromised during sepsis (Figure 2). The overall proportion of splenic and tissue specific B cells decreases, and antigen-specific antibody production is compromised [93]. Studies using mouse CLP models of sepsis have shown that drugs, such as Tubastatin A, which is a selective inhibitor of histone deacetylase 6, restore B cell counts during sepsis and have an added effect of increasing the number of innate immune cells, such as macrophages and neutrophils [94]. However, the direct interactions between these cell types that trigger their expansion is unknown and requires further investigation. Tubastatin A improves survival in mouse models of sepsis, however, the underlying mechanism for this drug in treating sepsis is also unknown.

## 4. Putative Therapies and Biomarkers Targeting Immune Suppression during Sepsis

In recent years, multiple studies have emphasised that immune suppression strongly correlates with sepsis outcome [4,50]. Studies have now shifted their focus in efforts to prevent immune cell dysfunction and cell death during sepsis as well as immune-enhancing therapies (Table 1) [100]. Using infectious animal models, it was shown that alveolar macrophages from granulocyte-macrophage colony stimulating factor (GM-CSF) deficient mice had a reduced ability to phagocytose pathogens [101]. Parallel studies using caecal ligation puncture (CLP) mouse models showed that treatment with recombinant GM-CSF reduced bacterial translocation and improved survival [102]. With this, investigators have tested stimulatory molecules such as IFN-γ, granulocyte colony-stimulating factor (G-SCF) and GM-CSF, in an effort to increase proliferation of immune cells during sepsis in the clinic. Studies utilising these factors in the clinic have showed a degree of effectiveness as these molecules acted as effective immune-stimulatory agents and did not exacerbate the hyper-inflammatory phase [103]. A clinical study looking at the effects of IFN-γ as an adjunctive immunotherapy revealed that IFN-γ appears to be well tolerated and improves the patient’s responses to infection during sepsis [104]. However, meta-analysis has revealed that GM-CSF and G-CSF did not show any sepsis-related survival benefit in the clinic [105]. Many factors impact survivability during sepsis since the disease is extremely heterogenous. Factors such as patient co-morbidities, steady-state immune cell populations as well as the causative pathogen, varies greatly amongst patients, therefore, one such treatment may not be effective in treating an entire population. As for GM-CSF and G-CSF therapy, meta-analysis does not take these factors into account. Indeed, GM-CSF therapy has shown clinical benefit in a subset of septic neonates who were primarily neutropenic. Therefore, proper diagnosis and combinatorial therapy seems to be the most viable option for treating sepsis when it comes to GM-CSF and G-CSF [106].

Recently, the role of IL-3 in sepsis pathogenesis has been investigated. IL-3 is known to stimulate the differentiation of hematopoietic stem cells into myeloid progenitor cells [107]. Studies have shown that IL-3 contributes to emergency myelopoiesis, triggering the expansion of monocytes and neutrophils during sepsis [108]. Consistent with these findings, others have shown that low levels of plasma IL-3 from sepsis patients correlated with poor survival [109]. On the contrary, it has been claimed that amplification of acute inflammation, triggered by IL-3, fuels the cytokine storm [46]. However, these findings have only been substantiated in limited mouse models of sepsis and it is well established now that emergency myelopoiesis is a critical event in human disease [110].

Lymphopaenia is another negative outcome of sepsis. Marked depletion of all lymphocyte populations, except for T_regs_, is evident in the circulation and tissues of sepsis patients [84].

In vivo studies using a sepsis two-hit model showed that IL-7 improved the host response and had a 92% survival during sepsis [111]. More importantly, clinical studies using CYT107 (recombinant IL-7) have shown reversal of the marked loss of CD4^+^ and CD8^+^ T cells following sepsis [112].

Contrary to IL-7, PD-L1 has been found to have potent effects in enhancing lymphocyte death during sepsis. Studies have found that dysfunction of T cells, NK cells, neutrophils and monocytes in both mouse models of sepsis, and in patients stricken with sepsis, was associated with PD-1 or PD-L1 expression [113]. Utilisation of blocking antibodies to inhibit the PD-1/PD-L1 axis has been shown to protect against sepsis-induced immune suppression, by partly inhibiting lymphocyte apoptosis and reversing monocyte dysfunction [114,115]. These findings collectively suggest that targeting and restoring immune function with immunomodulatory molecules could improve sepsis outcome for critically ill patients.

**Table 1:** Putative treatment strategies that aim to reduce immunosuppression during sepsis.

Treatment	Target & Action	Reference
GM-CSF	Increase activity and production of innate immune cells i.e., neutrophils	[116]
G-CSF	Increase innate immunity	[117]
IFN-γ	Increase leukocyte activity	[104]
IL-3	Induction of myelopoiesis	[46]
IL-7	Promotes proliferation and survival of lymphocytes	[112]
IL-15	Maintenance of NK cell population	[118]
Broad spectrum IgG	Regulate pro- and anti-inflammatory processes	[119]
Anti-PD1/PD-L1	Reduce cell death and promote T-cell responses	[120]
Anti-CTLA-4	Reduce cell death and promote T-cell proliferation	[121]

Sepsis biomarkers can be strong diagnostic tools, which help clinicians rapidly identify disease to treat patients promptly and appropriately (Table 2) [122]. Neutrophils have important roles in the hosts’ defense against infections. It has been shown that expression of CD64 on the surface of neutrophils dramatically increases during the early stages of sepsis, which returns to basal level after infection is resolved. Meta-analysis has shown that CD64 is a suitable marker for diagnosis of microbial infection during early disease pathogenesis [123]. Similar studies have determined that a combination of markers such as CD64, c-reactive protein (CRP) and procalcitonin (PCT) have profound prognostic value, which can accurately discriminate sepsis in critically ill patients [124]. Other markers such as CD25, a receptor for IL-2 expressed on T_regs_, also showed diagnostic capacity in the clinic [125]. It was found that higher expression of CD25 in the plasma of patients correlated with a higher rate of death [126]. It was also revealed that patients with sepsis had increased levels of soluble CD25 compared to other critically ill patients [127]. Other soluble markers such as binding immunoglobulin protein (BiP), which is released by stimulated macrophages, has been shown to be elevated in serum taken from septic patients [128].

Among all the notable markers, HLA-DR has been the most heavily investigated. It was reported over three decades ago that the expression of HLA-DR correlates with the progression of sepsis [129]. Specifically, the low expression of HLA-DR is considered to be a robust predictor of sepsis prognosis in critically ill patients [130]. Recently, an observational cohort study found that monocyte dysfunction was accompanied by significantly lower HLA-DR expression in non-survivors of sepsis [131]. While the aforementioned markers are generally reliable in theory, practical application remains an issue. Measuring the abundance of HLA-DR in the clinic could have some drawbacks, as cell staining must be performed, which is a lengthy process [132]. However, this issue could potentially be overcome with the optimisation and use of simpler quantitative measures such as quantitative polymerase chain reaction (qPCR).

**Table 2:** Putative diagnostic and/or prognostic biomarkers of sepsis.

Biomarker	Prognostic or Diagnostic	Reference
CD64	Diagnostic	[123]
CD25	Diagnostic	[125]
IL-6	Both	[133]
MCP-1	Both	[134]
CRP	Both	[135]
PCT	Both	[136]
BiP	Prognostic	[128]
PTX-3	Diagnostic	[137]
HLA_DR	Diagnostic	[63]
Calprotectin	Diagnostic	[138]
sTREM-1	Diagnostic	[139]

## 5. Conclusions

Sepsis remains as one of the deadliest diseases word-wide, yet, very little is known about the mechanisms underlying inflammation and immunosuppression associated with this disease. Based on past failed clinical trials that specifically targeted pathways affecting inflammation, it is clear that immune activation and inflammation is required to fight infection during sepsis and blocking inflammation could have a deadly impact. Understanding and developing therapies to improve immune system homeostasis and survival is now the new route for evolving therapies against this disease. Strategies focused on immunomodulating and immuno-stimulating therapies show great promise in preclinical studies and they stand as putative candidates for the treatment of sepsis for future clinical trials.

## Data Availability

Not applicable.
